# The molecular mechanisms in prenatal drug exposure-induced fetal programmed adult cardiovascular disease

**DOI:** 10.3389/fphar.2023.1164487

**Published:** 2023-04-20

**Authors:** Ting Wu, Kaiyu Zhou, Yimin Hua, Wen Zhang, Yifei Li

**Affiliations:** ^1^ Key Laboratory of Birth Defects and Related Diseases of Women and Children of MOE, West China Second University Hospital, Sichuan University, Chengdu, China; ^2^ Department of Ultrasonic Medicine, West China Second University Hospital, Sichuan University, Chengdu, China; ^3^ Department of Pediatrics, West China Second University Hospital, Sichuan University, Chengdu, China

**Keywords:** prenatal drug exposure, programmed diseases, cardiovascular disorders, molecular mechanisms, epigenetic regulation

## Abstract

The “developmental origins of health and disease” (DOHaD) hypothesis posits that early-life environmental exposures have a lasting impact on individual’s health and permanently shape growth, structure, and metabolism. This reprogramming, which results from fetal stress, is believed to contribute to the development of adulthood cardiovascular diseases such as hypertension, coronary artery disease, heart failure, and increased susceptibility to ischemic injuries. Recent studies have shown that prenatal exposure to drugs, such as glucocorticoids, antibiotics, antidepressants, antiepileptics, and other toxins, increases the risk of adult-onset cardiovascular diseases. In addition, observational and animal experimental studies have demonstrated the association between prenatal drug exposure and the programming of cardiovascular disease in the offspring. The molecular mechanisms underlying these effects are still being explored but are thought to involve metabolism dysregulation. This review summarizes the current evidence on the relationship between prenatal drug exposure and the risk of adult cardiovascular disorders. Additionally, we present the latest insights into the molecular mechanisms that lead to programmed cardiovascular phenotypes after prenatal drug exposure.

## Introduction

An optimal intrauterine environment is critical to maintaining fetal development. At the very beginning, harmful regents have been identified to be related to significant birth malformations, especially neurological and cardiovascular birth defects. Researchers have made enormous efforts to reveal the definite association between maternal drug administration and congenital heart diseases, which promoted the application of the FDA pregnancy category of drugs in clinical practice. With the rapid development and widespread concept of the “developmental origins of health and disease” (DOHaD) hypothesis, the prenatal environment shapes the health condition in adulthood, which has been discussed in the literature and published in the most recent 20 years. Pregnant exposure to smoking and smoking cessation pharmacotherapies would induce postnatal cardiovascular consequences, including disorders in blood pressure, endothelium-dependent relaxation, and vascular contraction (Kim et al., 2022). As a result, maternal nicotine intervention adversely affects the offspring’s cardiovascular outcomes. Moreover, a series of cardiometabolic disorders have been proven to be induced by a programmed process in the long-term developmental stage. Thus, it is believed that the adverse effect *in utero* would contribute to adulthood pathogenesis in the cardiovascular system. As a result, the ratio of maternal medication administration has risen to a higher level. A retrospective study on more than 19,000 women from 1976 to 2008 showed that about 90 percent of women took at least one prescription or over-the-counter drug during pregnancy. About 7.5% of pregnant women were exposed to antidepressants, and 65% of pregnant women had used acetaminophen during pregnancy, while ibuprofen and pseudoephedrine were also used by approximately one in six women. An increased number of pregnant women have been diagnosed positive for HIV; more than 8,000 HIV-positive women give birth each year in the United States, and 4,000 children are estimated to be exposed to antiretroviral drugs *in utero*. Medication safety during pregnancy administration has always been brought to attention, and evaluation of the safety of maternal treatment is a critical issue for perinatal management. However, there is limited information on the potential risks of offspring for most types of medication. Moreover, it is also important to underline the effects of maternal drug exposure on adult-programmed diseases.

**FIGURE 1:**
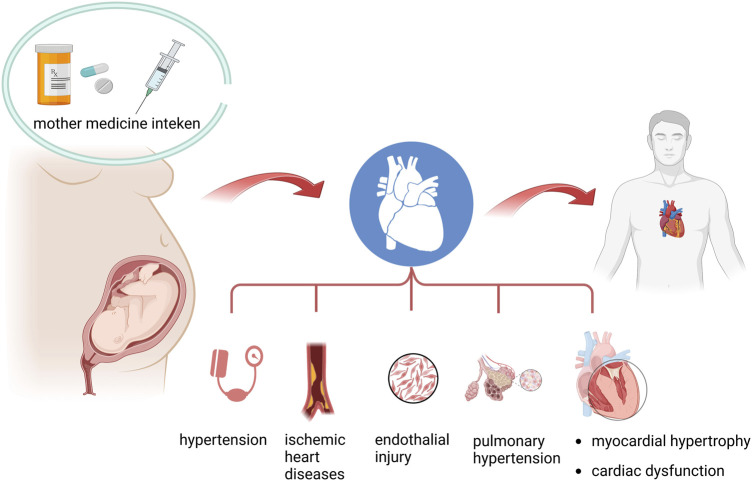
Summary of the association between prenatal drug exposure and programmed adult cardiovascular diseases.

The emerging evidence documented that once the fetus is exposed to certain drugs *in utero*, it would not only lead to impaired fetal growth patterns but also result in programmed alternations in cardiovascular function, metabolic syndrome, and neurodevelopmental disorders in adult life. Epidemic observations and experimental animal research demonstrated that exposure to glucocorticoids, antibiotics, antidepressants, antiepileptics, and other toxins in pregnant women leads to programmed cardiovascular diseases in adult offspring. Additionally, the molecular mechanisms are involved in metabolism dysregulation post-prenatal drug exposure. Herein, we summarized the evidence on the association between the risk of programmed adult cardiovascular disorders and prenatal drug exposure. Also, we presented the latest molecular mechanisms inducing programmed cardiovascular phenotypes (Figure 1).

## Association between prenatal drug exposure and programmed cardiovascular diseases/

Prenatal drug exposure, especially the administration of maternal medication, could complete transplacental transportation across the maternal–fetal barrier. Usually, the fetal circulating concentration of drugs would be much lower than the maternal side. However, even minimal drug exposure induced identical adverse fetal developmental outcomes. Meanwhile, several transmembranous proteins are involved in the regulation of drug trafficking between maternal and fetal circulation. ATP-binding cassette (ABC) transporters contribute dominantly to the drugs’ transplacental transportation, including p-glycoprotein, multiple drug-resistant protein, and breast cancer resistance protein. Several research studies demonstrated that the expression of ABC transporters is associated with the prevalence of cardiac birth defects (Wang et al., 2013; Wang et al., 2014a; Wang et al., 2014b; Wang et al., 2015; Wang et al., 2021; Tang et al., 2023).

### Glucocorticoids

Synthetic glucocorticoids are widely used to manage women at risk of preterm birth or who have undergone autoimmune diseases. However, the off-target adverse effects from exogenous glucocorticoids have been recorded (Teulings et al., 2020). Evidence suggested that excessive fetal glucocorticoids would cause abnormal skeletal muscle development and cardiovascular function both in the prenatal and postnatal phases. The impact of prenatal glucocorticoids would continue over the course of a person’s life (Liu et al., 2021). Convinced researchers documented that prenatal steroids would result in intrauterine growth restriction (IUGR). A “catch-up” that followed IUGR was considered an independent risk factor for hypertension and ischemic heart diseases in later life (Abrantes et al., 2020). Exposure to DEX *in utero* appears to impact heart growth and metabolic function adversely, but it is not associated with a significant loss of cardiomyocytes in adult mice (O'Sullivan et al., 2013). The adverse effect leading to a widened pulse pressure may be indicative of altered vascular compliance. While Teulings et al. (2020) showed that embryos exposed to DEX were growth-restricted and showed systolic and diastolic dysfunction, with an increase in cardiomyocyte volume but decreased cardiomyocyte nuclear density in the left ventricle. Madhavpeddi et al. (2022) demonstrated the involvement of angiotensin II in sex-selective cardiovascular function and autonomic changes in adult offspring exposed to DEX during the last 4 days of gestation in rats, which influenced the heart rate, heart rate variability, and mean arterial pressure. Murray et al. (2022) indicated that prenatal exposure to DEX would affect circadian rhythm gene expression and impair the related molecular oscillators. Prenatal betamethasone therapy accelerates lung development in preterm infants but may induce early programming events with long-term cardiovascular consequences. The activity of angiotensin-converting enzyme 2 (ACE2) was threefold higher than controls, which strengthens the association between prenatal glucocorticoid exposure and hypertension in offspring. Turner et al. (2017) suggested that an altered tubuloglomerular feedback response could be an underlying renal mechanism contributing to the development of hypertension in the DEX model of fetal programming. The lower tonic level of NO production in these DEX-exposed offspring may contribute to the development of hypertension as adults. Also, excessive glucocorticoid exposure during pregnancy elevated the susceptibility of the offspring’s heart to “second strike injuries,” such as ischemic/perfusion injuries (Peng et al., 2018).

Across pregnancy, maternal serum cortisol levels increase up to 3-fold (Stoye et al., 2020). In addition, the elevated glucocorticoid signaling *in utero* following prenatal stress was considered the critical mediator in transferring adverse effects to offspring (Krontira et al., 2020). Moreover, endogenous glucocorticoids would also contribute to adult programmed cardiovascular disorders. The maternal stress stimulation induced excessive glucocorticoid accumulation and increased the risk of long-term adverse cardiometabolic outcomes (Eberle et al., 2021). Severe obesity during pregnancy induced significantly increased endogenous glucocorticoid expression, which increased the mRNA levels of 11β-HSD2, NR3C1-α, and IGF2-1 in the placentas of females (Mina et al., 2015).

### Sex manner

Prenatal DEX exposure induced developmental toxicities in the cardiovascular system in offspring and changed metabolic hemostasis. However, the metabolic profiles presented a gender difference. Prenatal DEX would increase blood pressure among male offspring. The primary differential metabolites induced by prenatal DEX exposure included lactic acid, carnitine, cortexolone, bile acid, phosphatidylcholine, uric acid, and platelet-activating factor, which may participate in dexamethasone multi-organ toxicities and multi-disease susceptibility (Chen et al., 2019a). Alhamoud et al. (2021) found that the phenotype was more frequently observed in male offspring due to increased levels of sodium and potassium and two-chloride cotransporter protein abundance in renal thick ascending limb.. However, female offspring were absent from such changes. In a rat experiment, Hsu et al. (2020) indicated that adult male offspring with prenatal DEX exposure and a postnatal high-fat diet presented obesity, increased systolic blood pressure, and peripheral and central insulin resistance. In addition, prenatal betamethasone exposure engenders male-specific impairment of sinoatrial node function and myocardial ischemia tolerance resulting from an increased adenosine metabolism in the heart (Kiguti et al., 2017). Furthermore, the different adrenal adaptations might be explained in a sex-specific manner. The reduced absolute adrenal weight had been observed by 12 months of age post intrauterine glucocorticoid exposure, and the ACTH receptor (Mc2R) was increased in a short time until sexual maturity in mice, which indicated that the adrenal gland dysfunction was supposed to exist in male offspring after maternal glucocorticoids exposure.

However, some programmed heart rate and metabolism changes had only been observed in female offspring. The elevated heart rate caused by excessive glucocorticoid exposure could only be reversed by exaggerated pressor and angiotensin II receptor blockade, which presented a sex-selective manner (Madhavpeddi et al., 2022). Moreover, sex-specific differences existed in the lipid metabolism of adult rats after DEX exposure *in utero*. The female offspring presented metabolic dysregulation of genes, while such impairment failed to be identified in males (Murray et al., 2021).

Moreover, although both males and females demonstrated dysregulation of blood pressure post prenatal betamethasone exposure, different molecular mechanisms varied between male and female offspring. Hendricks et al. (2018) found that female offspring exhibited lower expression of the PI3K pathway, while male offspring presented activation of MAPK/ERK kinases.

### NSAIDs

Analgesia during pregnancy is often necessary. Due to their easy availability, over-the-counter (OTC) analgesics, mainly involving non-steroidal anti-inflammatory drugs (NSAIDs), have been widely used in pregnant women. Such analgesic medications and their metabolites demonstrated the capability to cross the placenta and influence fetal development (Antonucci et al., 2012). However, several studies revealed the association between intrauterine NSAID exposure and birth defects. Several recent studies have observed the impact on the onset of long-term cardiovascular disease. Several drug transporter proteins were expressed in the placental barrier membranes, including apical and basal membranes, which regulated the fetal exposure of drugs and their substrates. The involved transporters could be either blocked or facilitated. The organic anion transporter (OAT) 1/SLC22A6 efflux transporter has been proven to mainly regulate the concentration of NSAIDs by transporting NSAIDs into fetal blood, while OAT4/SLC22A11 also contributed to facilitating the import of NSAIDs to fetal circulation. According to the documents of the FDA, United States, paracetamol was recommended in pregnancy for either fever or pain relief. However, aspirin and ibuprofen had been recommended for administration during pregnancy, although fetal paracetamol exposure had been considered to be associated with fewer risks. Nevertheless, previous studies demonstrated a causal relationship between maternal paracetamol use during pregnancy and fetal ductus arteriosus constriction or birth defects (Källén, 2014; Interrante et al., 2017; Bateman et al., 2021). Moreover, the same finding was confirmed by Aker et al., which indicated that using NSAIDs in the third trimester would slightly increase the risk of cardiovascular defects (Zafeiri et al., 2021). Marsh et al. (2014) provided more detailed results on the association between prenatal NSAID use and specific types of cardiac malformation. They exhibited that only two types of related malformations had been identified with NSAID pregnant use: tetralogy of Fallot with maternal acetaminophen use (OR = 1.6; 95% CI = 1.1–2.3) and dextrotransposition of the great arteries with intact ventricular septum with maternal general NSAID use (OR = 3.2; 95% CI = 1.2–8.7). In addition, the risk for pulmonary valve stenosis, hypoplastic left heart syndrome, and tetralogy of Fallot was found to be higher in pregnancies with the consumption of paracetamol than NSAIDs. Fikret et al. (2015) demonstrated that a reduced number of cardiomyocytes and cellular size in the heart after prenatal diclofenac sodium exposure resulted in a decreased volume of ventricular walls. The loss of the essential number and size of cardiomyocytes was associated with adult heart failure and cardiac fibrosis. In addition, Zengin et al. (2013) indicated that diclofenac sodium application decreased the mean volume fraction of tunica media in all vessel walls. Elastic fibers of the vessel wall were affected by diclofenac sodium treatment, as a decrease in the elastic fiber was observed in prenatal diclofenac sodium-treated mice.

However, some studies presented opposite opinions on the prenatal administration of NSAIDs, even though some results demonstrated long-term advantages for the cardiovascular system. For example, Novi et al. (2021) demonstrated that intrauterine paracetamol exposure failed to impair the thoracic aorta reactivity to acetylcholine in both male and female offspring. Moreover, insulin sensitivity, adipose tissue deposition, and blood pressure presented no difference between exposure and control groups. So, it concluded that program vascular and metabolic alternations were not related to prenatal paracetamol exposure (Novi et al., 2021). Also, Chen et al. (2019b) revealed that intrauterine exposure to aspirin mediated the postnatal long-term blood pressure regulation in childhood, indicating a benefit to preventing high blood pressure by a decrease of 0.62 mmHg for systolic blood pressure and 1.04 mmHg for diastolic blood pressure.

### Antidepressants

Antidepressant use during pregnancy increases the risk of several adverse effects (Domar et al., 2013). Moreover, several studies proved that using antidepressants during gestational age would induce birth defects, especially for congenital heart disease. For example, Sun et al. (2022) indicated that the use of maternal antidepressant 3 months before pregnancy and early pregnancy exposure to an antidepressant would elevate the risk of congenital heart disease among the offspring. In addition, intrauterine fluoxetine exposure was associated with several congenital malformations (RR = 1.18, 95% CI = 1.08–1.29). However, the associated risk was negligible, and convinced evidence confirmed such an elevation in the risk of malformations. Moreover, the cardiovascular defects (RR = 1.36, 95% CI = 1.17–1.59), especially the septal defects (RR = 1.38, 95% CI = 1.19–1.61), indicated a higher risk ratio among newborns after fluoxetine exposure (Gao et al., 2017). Moreover, genetic variants also contributed to determining the associated risk of cardiac birth defects. Cytochrome P450 genetic polymorphisms have been proven to be involved in regulating maternal drug side effects on embryonic heart development. Additionally, the pregnant use of benzodiazepines did not appear to be associated with congenital malformations. While the combination of benzodiazepines and selective serotonin reuptake inhibitors (SSRIs) leads to a higher risk of congenital malformations, indicating a caution of the combination of medication administration during pregnancy. However, no evidence compared the relative risk of such a combination with SSRIs alone, so it could not determine the increased risk of SSRIs or the collaboration function of benzodiazepines and SSRIs (Grigoriadis et al., 2019).

Current evidence demonstrated that SSRIs were associated with mildly increased adverse effects on preterm delivery, persistent pulmonary hypertension of the newborn (PPHN), and neonatal intensive care unit admissions (Lebin and Novick, 2022). A systematic review from Fitton et al. (2020) also confirmed the association between prenatal antidepressant exposure and developmental disorders. Another meta-analysis from Uguz et al. emphasized the adverse effects of pregnant women using antidepressant medications on neonatal outcomes, including preterm birth, low birth weight, and a higher risk of PPHN. Although exposure to SSRIs during pregnancy increased the risk of PPHN, the calculated risk ratios were various among different types of SSRIs. Finally, Masarwa et al. (2019) carried out a network meta-analysis. They demonstrated that sertraline was ranked as most likely to have the lowest risk for PPHN among other SSRIs, suggesting that it would have the best safety profile for use in pregnancy in this regard.

Although several studies indicated the association between antidepressants and birth defects, limited data revealed the long-term outcomes in adulthood, especially in the cardiovascular system. The recurrent evidence found that maternal exposure to antidepressants brought side effects in aorta reactivity and nitric oxide metabolites. Marques et al. (2017) confirmed that prenatal fluoxetine exposure blunted the aortic adaptive response in the offspring, indicating a reduction in aortic contraction in the aorta. Previous research has drawn a close relationship between maternal depression and offspring’s high risk of cardiovascular diseases. So, antidepressant administration during pregnancy should be considered with all advantages and disadvantages. So, several efforts have been made to attenuate the side effects of antidepressants. Fluoxetine exposure induced impairment of NADPH oxidase and mitochondrial biogenesis in the hearts of the mice model of depression, while using suberoylanilide hydroxamic acid (SAHA) helps restore the expression of PGC-1a and Nrf2 to rebuild normal mitochondrial function, partially preventing programmed cardiovascular diseases (Higashi et al., 2016). ASD is one of the most common and highly heritable neurodevelopmental diseases, affecting 1%–2% of children under the age of three. Although studies have implicated genetic predispositions, environmental risk factors, and maternal depression as the pathophysiology of ASD, it remains unclear. The association between antidepressant usage during pregnancy and the likelihood of ASD in children has been established. A systematic review from Mathew et al. (2022) demonstrated that maternal antidepressant usage during pregnancy would elevate the risk of neurodevelopment disorder among offspring. Moreover, paternal antidepressant administration would also cause a higher risk of autism in offspring. In addition, this research documented that even maternal pre-conceptional antidepressant use would also inherit adverse outcomes. So, prenatal antidepressant exposure would result in multiple system dysfunction lifelong.

### Antiepileptic

Epilepsy during pregnancy is associated with increased risks of adverse pregnancy and perinatal outcomes. Bromley et al. (2017) performed an observational study and found that antiepileptic drugs were associated with cardiac malformations. Moreover, a systematic review from Tanoshima et al. identified that the use of valproic acid (VPA) increased the risk of cardiac malformations by 2–7 fold and that valproic acid should be avoided as first-line therapy during pregnancy. However, Razaz et al. (2017) demonstrated that antiepileptic drug use during pregnancy is generally not associated with adverse outcomes. They concluded that all the adverse outcomes were inherited from the epilepsy condition. Valproic acid administration during pregnancy leads to an alternating metabolomics profile. The impaired metabolism in offspring resulted in programmed neurodevelopment disorder and cardiovascular dysfunction. Previous research indicated that prenatal VPA exposure induced congenital heart diseases. In addition, exposure to VPA also increases the risk of coronary artery disease by inhibiting the proliferation of human coronary vascular cells (Kim et al., 2022). However, the use of VPA post-infarction demonstrated a cardio-protective role in the prevention of heart failure development.

### Antibiotics

Antibiotic residues in the environment have been shown to induce magnificent adverse effects on the developmental process and lead to birth defects. Moreover, a series of studies underlined the involved molecular mechanisms. Beyond the effects on birth defects, the antibiotic residue would also cause long-term dysfunction in the cardiovascular system through cross-generational cardiotoxicity. Xuan et al. (2022) performed *in vivo* research to reveal the mRNA–miRNA network regulation involved in cross-generational cardiotoxicity after prenatal antibiotic exposure. Let-7a-5p had been identified as the canonical pathway regulating associated cardiac hypertrophy, which mediated the cross-generational cardiotoxicity of antibiotics in zebrafish (Xuan et al., 2022).

Macrolides such as azithromycin are commonly prescribed antibiotics during pregnancy. The good oral bioavailability and transplacental transfer (3%) of azithromycin make this drug suitable for treating sexually transmitted diseases, toxoplasmosis, and malaria (Antonucci et al., 2022). However, the debates on the effects of administering azithromycin during pregnancy on offspring outcomes are ongoing. Fetal and neonatal outcomes following prenatal azithromycin exposure have been investigated by several studies, yielding conflicting results. Miscarriage, major malformations, and cardiovascular malformations were reported as the dominant adverse outcomes in previous observational studies. A systematic review demonstrated that azithromycin use during pregnancy was significantly associated with an increased risk of major gastrointestinal or musculoskeletal malformations compared to other antibiotics (penicillin or cephalosporins). However, the Slone Epidemiology Center Birth Defects Study from 1994 to 2008 declined the association between the use of macrolides (erythromycin, azithromycin, and clarithromycin) and the risk of congenital heart diseases. Although a slightly increased ratio of cardiovascular malformations had been addressed among the offspring exposed to azithromycin (1.6% Vs. 0.9%), this failed to reach a significant difference. Furthermore, a nationwide, register-based cohort study was performed in Denmark between 1997 and 2016. Among 1,192,539 live-birth pregnancies, 13,019 pregnancies were treated with macrolides and were compared with those in which penicillin was used (51,515) or no antibiotic was given (995,673), which presented that the use of macrolide antibiotics during the first trimester of pregnancy was not associated with an increased risk of major birth defects. However, the long-term effect of prenatal exposure to macrolides only focused on several allergic disorders, including asthma, and the effects on the cardiovascular system were missing. So, currently, there is no conclusive evidence to support the claim that azithromycin use by pregnant women causes adverse outcomes in their offspring.

Metabolic disorders appearing in later life are also suspected to reflect changes in early programming. Additionally, recent research demonstrated that maternal antibiotic use could alter the offspring’s gut microbiota, resulting in changes in short-chain fatty acids (SCFAs) and related metabolites. Minocycline administration reduced plasma acetic acid levels, which induced programmed hypertension (Hsu et al., 2022). However, prenatal amoxicillin exposure could lower blood pressure in adults by reshaping the offspring’s microbiota (Galla et al., 2020). In another study, amoxicillin prenatal exposure increased colonic digest alkaline phosphatase (AP) and TLR2- and TLR4-stimulant concentrations in rectal digest in adult offspring, which altered the interaction between gut microbiota and immune responses (Arnal et al., 2015).

### Antiretroviral therapy

With the increased number of HIV-infected women, the effects of antiretroviral therapy during pregnancy among HIV-positive mothers have been studied, especially for the potential adverse effects on offspring. Globally, over 1.4 million pregnant women are living with HIV. Antiretroviral therapy management during pregnancy helps prevent mother-to-child transmission of HIV. However, mitochondrial damage had been observed in some offspring. The combinations of zidovudine/lamivudine/abacavir caused the mitochondrial reserve capacity oxygen consumption rate in bone marrow mesenchymal fibroblasts in 3-year-old patas offspring (Liu et al., 2016). In addition, antiretroviral therapy-exposed patas offspring showed a compensatory increase in heart mtDNA and a 50% loss of brain mtDNA at 1 year of age. However, mitochondrial morphological damage and mtDNA loss were persistent in blood cells of antiretroviral therapy-exposed infants up to 2 years of age and in the heart and brain of antiretroviral therapy-exposed patas up to 3 years of age (the human equivalent of 15 years) (Poirier et al., 2015). Moreover, evidence suggested that HIV-infected adults on antiretroviral therapy have a higher prevalence of hypertension when compared with HIV-uninfected individuals. At the same time, antiretroviral therapy would cause direct endothelial injury and promote endothelial dysfunction, an independent risk factor of cardiovascular diseases. Gois et al. (2015) used an experimental model to show that maternal exposure to tenofovir during gestation results in the over-activation of the renin–angiotensin–aldosterone system (RAAS), upregulation of renal sodium transporters, and hypertension in the offspring.

Fetoplacental vascular endothelial dysfunction was thought to be at the origin of chronic diseases such as diabetes and obesity later in life. As HIV and antiretroviral therapies were found to be associated with endothelial dysfunction and change in the intrauterine environment, children exposed *in utero* to these conditions were considered to be at greater potential risk of programmed cardiovascular diseases by epigenetic regulation (Nkeh-Chungag et al., 2021). An *in vivo* experiment confirmed that the administration of combination antiretroviral therapy was associated with poor placental insertion and development of IUGR. Indeed, poor placental function has been proven to be associated with obesity, hypertension, and diabetes in later adult life. While García-Otero et al. (2022) demonstrated an observational study to identify that intrauterine exposure to HIV and antiretroviral therapy were not associated with cardiovascular changes from fetal development to infant growth, they did not measure the long-term outcomes of the cardiovascular system.

### Tetrahydrocannabinol

Notably, cannabis has been widely used across several countries, and the effect of delta-9-tetrahydrocannabinol (THC) is impaired fetal development, leading to several adult-programmed diseases. It is estimated that approximately 20% of pregnant women use cannabis during pregnancy. Exposure to cannabis-induced placental dysfunction was associated with postnatal metabolic diseases (Lee et al., 2021; Lee and Hardy, 2021). Martínez-Peña et al. (2022) indicated that intra-uterine THC exposure unregulated the expression of miR-122-5p, which inhibited insulin-like growth factor 1 receptor (IGF1R). IGF1R has been associated with cardiovascular diseases. The IGF1R inhibitor caused cardiac contractile dysfunction. In addition, IGF1R participated in myocardial remodeling after the myocardial infarction, which was regulated by miR-223-3p. Moreover, the enhanced suppression of the IGF1R-PI3K-AKT pathway would induce further apoptosis of cardiomyocytes under AngII stimulation. Furthermore, the attenuated IGF1R impaired mitochondrial quality control and programmed cardiometabolic disorders. Cannabis exposure during pregnancy would also result in intrauterine growth restriction (IUGR), and the significant IUGR indeed induced cardiovascular diseases in adulthood. Researchers believe the significant effects on programmed cardiovascular diseases are mainly inherited from IUGR. However, a recent study from Lee et al. demonstrated that long-term exposure to THC, the dominant component of cannabis, induces greater left ventricular anterior wall thickness and impairment of cardiac output function by increased collagen I and III expression, demonstrating an independent pathway from IUGR (59).

## Molecular mechanisms involved in prenatal drug exposure-associated cardiovascular disorders

### Glucocorticoid regulation

The literature has long discussed the notion that the prenatal environment contributes to health in adulthood (Krontira et al., 2020). A plethora of studies has associated an adverse intrauterine environment with negative cardiovascular, metabolic, endocrine, and psychiatric outcomes. Extensive epidemiological studies have definitively documented the negative impact of prenatal stress. A major biological mechanism is involved that has been implicated in mediating risk trajectories following prenatal stress, namely, elevated glucocorticoids. There are two kinds of glucocorticoid receptors: the glucocorticoid and mineralocorticoid receptors (GR and MR, respectively). In fact, high prenatal glucocorticoid signaling has been shown to influence the development of specific organs. There was less evidence to suggest that elevated endogenous maternal glucocorticoids directly mediate the effects of prenatal stressors. Nonetheless, animal studies strongly suggested that maternal glucocorticoids are relevant to the adverse effects of prenatal stress on offspring.

Due to the barrier function of the placenta, the glucocorticoids in fetal compartments reached less than 10% of the concentration in maternal plasma. Such a difference was regulated by the 11β-HSD2, which helps the fetus resist the harmful glucocorticoid transfusion. The 11β-HSD2 is located in the fetal side membrane of the placenta and is involved in catalyzing the transferred active cortisol (Shearer et al., 2019). However, 11β-HSD2 failed to regulate the synthetic glucocorticoids, including dexamethasone and betamethasone, which were not the substrates of 11β-HSD2. So exogenous exposure during pregnancy usually induces more adverse impact in offspring than endogenous ones. Prenatal restraint stress impaired placental growth and vascularization. In addition, the elevated cortisol reduced the expression of *Vegf*, *Crhr1*, *Nr3c1*, and *Hsd11b2* (Gross et al., 2018; Shearer et al., 2019). Additionally, the elevated glucocorticoids participated in the epigenetic regulation of particular genes associated with programmed cardiovascular diseases. GR activation resulted in stable DNA demethylation of cytosine in glucocorticoid response elements in the promoter of the liver-specific tyrosine aminotransferase (*Tat*) gene (Gross et al., 2018). Provençal et al. (2020) reported that progenitor cells were treated with dexamethasone *in vitro* during differentiation, changes in DNA methylation were identified, and the solid remained for more than 3 weeks. The CpG sites manifesting the DNA methylation changes were enriched for bivalent or poised enhancer or promoter regions.

**FIGURE 2:**
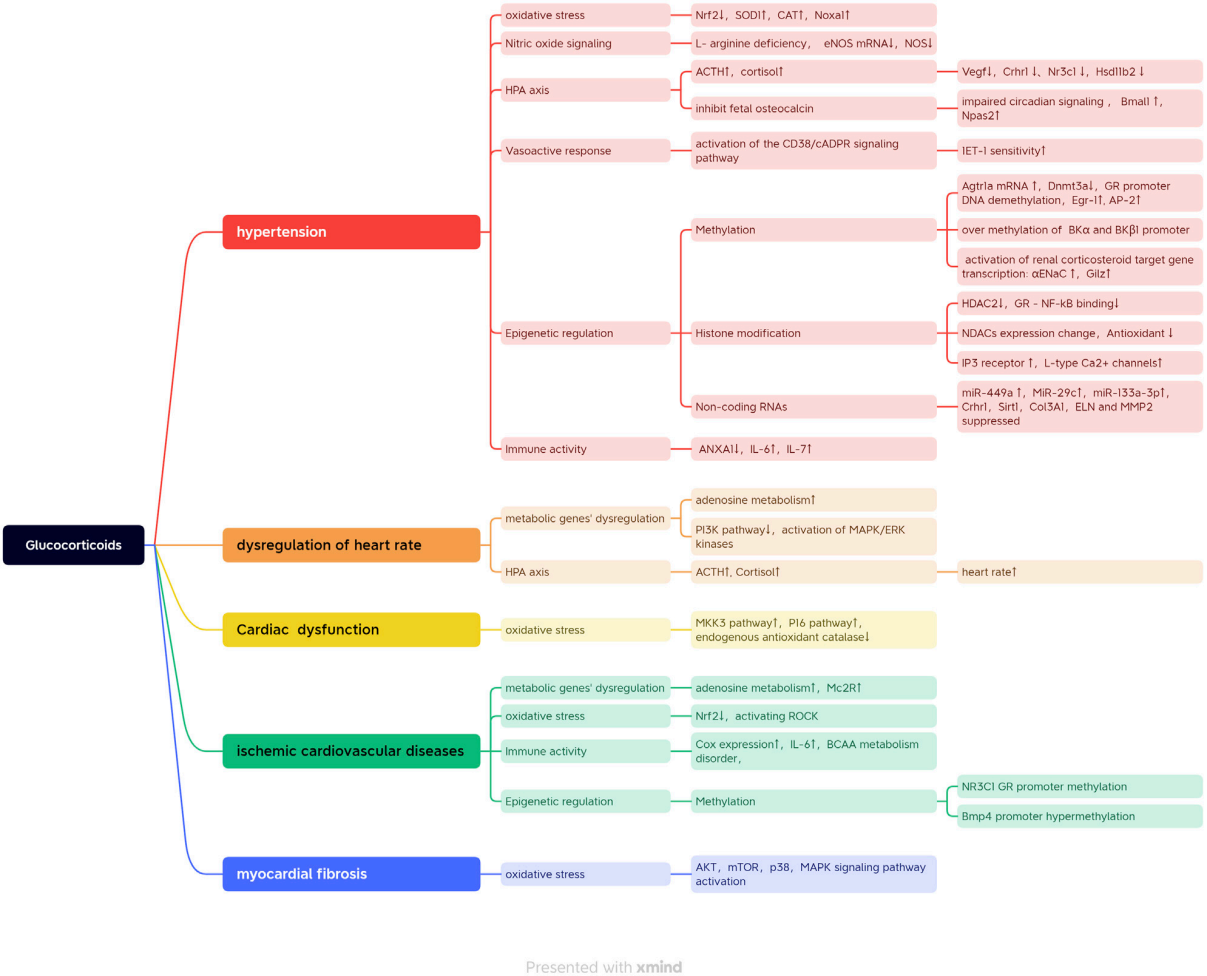
All the regulation mechanisms of glucocorticoid involved in programmed cardiovascular diseases.

On the other hand, the GR was also mediated by CpG methylation post prenatal stress and functionally regulated the sites of cAMP-response elements (CREs) at −4408 and −3896, and Sp1 binding sites at −3425 and −3034 were identified at the GR untranslated exon one promoter in fetal stress programmed ischemic myocardial injuries (Xiong et al., 2016). Fetal prednisone administration increased the level of cortisol in offspring (de Steenwinkel et al., 2014). Nguyen et al. presented that the increased phenylethanolamine N-methyltransferase gene expression *via* the altered transcriptional activity of Egr-1, AP-2, and glucocorticoid receptor (GR) was a possible mechanism for programmed hypertension later in life (Sheen et al., 2015). The loss of estrogen caused a reduction in the binding of GR to related elements at the promoter region of Agtr1 and Agtr2, resulting in decreased expression of Agtr1 and increased expression of Agtr2 in the myocardium (Xue et al., 2015). Thus, the persisting expression of a high estrogen level in the female heart contributed to the protective role of ischemia and reperfusion injury (Xue et al., 2015). All the regulation mechanisms of glucocorticoids involved in programmed cardiovascular diseases are summarized in Figure 2.

### Oxidative stress

Hypertension can originate from early-life exposure to glucocorticoid exposure associated with oxidative stress. Rodent studies found that continuous postnatal oxidative stress was related to promote cardiovascular aging, resulting in myocardial or vascular dysfunction in heart failure and hypertension (Zambrano et al., 2021). Moreover, the postnatal high-fat diet exacerbated systematic oxidative stress post-prenatal DEX exposure, which accelerated the process of programmed hypertension (Hsu et al., 2018). Nuclear factor erythroid-derived 2-related factor 2 (Nrf2) had been inhibited by prenatal DEX administration, which was involved in the regulation of oxidative stress-induced damage and autophagy. Meanwhile, the supplementary of dimethyl fumarate (DMF) would activate Nrf2 signaling and protect the offspring from programmed hypertension (Hsu et al., 2019). Lin et al. (2018) demonstrated that DMF particularly increased renal expression of Nrf2, reducing systematic oxidative stress by decreasing dimethylarginine and activating genes related to nutrient sensing and autophagy (*Pparb*, *Pparg*, *Ppargc1a*, *Ulk1*, and *Atg5*). Lamothe et al. (2021) suggested that glucocorticoids may mediate the fetal programming of hypertension by downregulating the capability of anti-oxidative stress *via* an epigenetic-mediated mechanism. The research demonstrated that programmed offspring displayed reduced antioxidant glutathione peroxidase 1 (Gpx1) expression, increased superoxide dismutase 1 (SOD1) and catalase (CAT) expression, and increased pro-oxidant NADPH oxidase activator 1 (Noxa1) expression in the adrenal glands. In addition, prenatal DEX exposure alters the expression of epigenetic regulators histone deacetylases (HDACs) in offspring. Prenatal nicotine exposure destroyed vascular reactivity, which led to uncontrolled blood pressure in adult male rat offspring in a sex-dependent manner. A molecular mechanism study proved that nicotine exposure significantly enhanced the expression of NADPH oxidase Nox2/gp91 and mediated the increased risk of hypertension in adulthood *via* a Nox2-dependent feature (Xiao et al., 2011).

### Nitric oxide signaling

Nitric oxide (NO) and hydrogen sulfide (H_2_S) pathways are involved in the development of hypertension. Therefore, prenatal DEX exposure would induce adult hypertension and a postnatal high-fat diet. However, maternal N-acetylcysteine therapy could restore the expression of genes of H_2_S-generating enzymes and reduce oxidative stress, which helps prevent programmed hypertension (Tai et al., 2016). Moreover, betamethasone induces increased Na^+^ uptake in the renal proximal tubule by suppressing nitric oxide, which would also contribute to the pathogenesis of hypertension (Su et al., 2015). In addition, Lu et al. (2016) demonstrated that the early postnatal treatment of 12-(3-adamantan-1-yl-ureido)-dodecanoic acid (AUDA) or 15-deoxy-Δ(12,14)-prostaglandin J2 (15d-PGJ2) would reduce the incidence of programmed hypertension secondary to prenatal DEX exposure. The beneficial effects of AUDA and 15d-PGJ2 therapy include inhibition of soluble epoxide hydrolase (SEH), increases in renal angiotensin converting enzyme-2 (ACE2) and angiotensin II type 2 receptor (AT2R) protein levels, and restoration of nitric oxide bioavailability. In addition, NO signaling also participated in endothelial regulation after antidepressant exposure. Higashi et al. demonstrated that maternal exposure to fluoxetine adversely affected aortic reactivity in adult offspring. Furthermore, they identified that early exposure to fluoxetine in the fetal stage impaired the aortic level of NO and COX-1 expression in a sex-specific manner, which enhanced endothelial modulation of aortic contractile response (Higashi et al., 2018; Grigoriadis et al., 2019).

### HPA axis

The negative hypothalamic–pituitary–adrenal axis regulation caused by glucocorticoid exposure in the fetus misled the activation of immune responses *via* the activation of Th17 cells and TLR4, which contributed to programmed cardiovascular diseases (Costa et al., 2022). Yadav et al. (2022) presented embryonic osteocalcin, determined the adrenal growth, adrenal steroidogenesis, blood pressure, and electrolyte equilibrium, and stimulated the expression of circulating corticosterone levels during the acute stress response in adult offspring. However, prenatal glucocorticoid exposure inhibited fetal osteocalcin and impaired the development of adrenal glands. Tharmalingam et al. (2020) demonstrated that glucocorticoid programmed adrenals have impaired circadian signaling and that changes in adrenal circadian rhythm were considered an underlying molecular mechanism responsible for the development of hypertension, with increased expression of *Bmal1* and *Npas2*.

The HPA axis still made a significant contribution to maintaining heart rate and blood pressure. However, prenatal glucocorticoid exposure showed an impairment of the HPA axis in offspring. In addition, in research on the hypoglycemia model, significant elevation in ACTH and cortisol (2∼3 folds) had been exhibited, which were associated with increased heart rate and systolic blood pressure (Schiffner et al., 2017).

### Gut microbiota

Gut microbiota-derived metabolites, particularly SCFAs and their receptors, were linked to hypertension. The antibiotics used during pregnancy negatively affected the offspring’s gut microbiota. Minocycline exposure decreased the abundance of the genera *Lactobacillus*, *Ruminococcus*, and *Odoribacter*. Additionally, minocycline leads to the reduction of plasma acetic acid and butyric acid levels. Minocycline exposure associated with hypertension was related to the increased expression of several SCFA receptors (Hsu et al., 2021). Moreover, individually or together, minocycline-induced hypertension was associated with the aberrant activation of the RAAS. In addition, Galla et al. (2020) demonstrated that amoxicillin exposure in early life was also associated with changes in gut microbiota and decreased blood pressure. The administration of amoxicillin reshaped the offspring’s microbiota with an alteration in the Firmicutes/Bacteriodetes ratio. They found that the decrease in blood pressure was due to the lowering of Veillonellaceae, which consisted of succinate-producing bacteria. Moreover, the elevated plasma level of succinate was identified to be associated with hypertension.

### Vasoactive response

Prenatal exposure to glucocorticoids increases cardiovascular risks related to vascular dysfunctions in offspring. Lee et al. (2014) demonstrated that prenatal glucocorticoid exposure increased the sensitivity of ET-1 and resulted in vasoconstriction. The increased response to ET-1 is mediated by the activation of the CD38/cADPR signaling pathway. In addition, DEX exposure did not alter the Ca^2+^/voltage-sensitivity of Ca^2+^-activated K^+^ channels but downregulated the expression of α and β1 subunits in both fetal and adult mesenteric arteries (Xu et al., 2020). Moreover, estrogen had been identified to be involved in regulating the abnormal expression of angiotensin II receptors. The results demonstrated that estrogen protected the maternal high-fat diet-mediated offspring’s cardiac hypertrophy, indicating a higher risk of cardiovascular remodeling among male offspring (Chen et al., 2021).

### Immune activity

Maternal stress or the administration of synthetic glucocorticoids to improve the survival chances of premature newborns is associated with a postnatal increased risk for immune diseases. In addition, the placental and fetal 11β-HSD2 would mediate fetal glucocorticoid exposure, altering the HPA axis function. The disorder of the HPA axis might impair fetal immune development, driving elevated risks of infection and autoimmune diseases (Solano et al., 2016). Zhao et al. (2022) demonstrated that gestational maternal immune activation in mice would induce persistent vascular dysfunction in the adult offspring *via* the increased expression of cyclooxygenase-2 (COX2). Moreover, a maternal infection triggered immune activation during pregnancy, and the activated immune response increased the offspring’s risk of developing a variety of metabolic disorders. The immune activity caused disorders of the metabolism of branched-chain amino acids (BCAAs), which was correlated with the concentrations of IL-6 and TNFa (Kowash et al., 2022). A previous study demonstrated that BCAA catabolic defect, which increased the concentration of BCAA, was considered a metabolic hallmark of a failing heart resulting from Krüppel-like factor 15 (KLF15)-mediated transcriptional reprogramming (Sun et al., 2016). Furthermore, Yang et al. (2015) conducted matched-pair case–control research and demonstrated that BCAAs were significantly associated with coronary artery disease. Furthermore, such a relationship was independent of diabetes, hypertension, dyslipidemia, and body mass index. Maternal virus infection and related antiretroviral therapy also altered fetal immune activity. Jacobsen et al. (2021) suggested that maternal influenza infection might impair immune ontogeny and increase susceptibility to early-life infections in offspring. Enhanced vulnerability to infection in neonates is associated with reduced hematopoietic development and immune responses. In particular, alveolar macrophages of offspring exposed to maternal influenza have a reduced capacity to clear second-hit pathogens. This impaired pathogen clearance is partially reversed by the adoptive transfer of alveolar macrophages from healthy offspring born to uninfected dams (Jacobsen et al., 2021). Evidence suggests that prenatal immune system disturbance contributes mainly to the pathophysiology of vascular disorders. The maternal immune activation induced oxidative stress and inflammatory changes, impaired the function of placental barrier permeability of pregnant rats, and caused endothelial dysfunction among offspring (Simões et al., 2018).

### Epigenetic regulation

#### Methylation

Mechanically, endothelial dysfunction has been proven to be involved in the development of hypertension. In addition, epigenetic regulation was identified to be associated with programmed endothelial dysfunction. In offspring of pregnant rats receiving a low-protein diet or dexamethasone, a synthetic glucocorticoid, mRNA expression of Agtr1a was upregulated, concurrent with reduced expression of DNA methyltransferase 3a (Dnmt3a), reduced binding of DNMT3a to the *Agtr1a* gene, and DNA demethylation (Kawakami-Mori et al., 2018). Agtr1a expression is epigenetically modulated by excessive glucocorticoids and leads to adult-onset salt-sensitive hypertension while decreasing DNMT3a binding and DNA demethylation at the Agtr1a locus (Kawakami-Mori et al., 2018). Dexamethasone-treated Agtr1a-deficient mice failed to show salt-induced BP elevation, despite reduced expression of Dnmt3a. The increasing DNA methylation of the GR promoter of NR3C1 elevated the atherosclerotic risk in a human monozygotic twin study. The low DNA methylation of the *GR*-*1C* gene would reduce the heart rate and cortisol levels. In addition, increased promoter methylations within BKα and BKβ1 post-DEX exposure were compatible with reduced expression of the two genes, which was associated with hypertension (Xu et al., 2020). Peng et al. (2018) found that excessive glucocorticoid exposure would lead to higher sensitivities to ischemic/perfusion injuries by causing long-term hypermethylation on the Bmp4 promoter. In addition, the absence of BMP4 protective effect on cardiomyocytes was responsible for more severe damage in ischemic/perfusion injuries. Additionally, the effects of prenatal glucocorticoids were transmissible to the second generation by epigenome alteration with more variable 5 mC levels, particularly at non-promoter loci (Cartier et al., 2018). Moreover, prenatal antibiotic exposure would also alter the methylation levels of specific genes. Vidal et al. (2013) reported an inverse association between *in utero* exposure to antibiotics and lower birth weight, which changed the methylation levels of the particular gene, including IGF2, PLAGL1, and MEG3. Moreover, epigenetic regulation might cause transgenerational impact on cardiovascular diseases. Dumeige et al. (2020) demonstrated that preterm birth is associated with mice’s epigenetic programming of transgenerational hypertension. They found robust activation of renal corticosteroid target gene transcription at birth in preterm mice (*αENaC* (+45%), *Gilz* (+85%)), independent of any change in mineralocorticoid or glucocorticoid receptor expression (Dumeige et al., 2020). The offspring of the preterm group displayed increased blood pressure in the second and third generations, associated with increased renal *Gilz* mRNA expression, despite similar MR or GR expression and plasma corticosteroid levels measured by LC-MS/MS. Furthermore, the methylation level of the *Gilz* promoter was reduced to promote its transcription. Moreover, the over-methylation of the 11β-HSD2 promoter was determined to significantly contribute to hypertension.

#### Histone modification

Histone deacetylation was also found to be a clue to induce what appears to be prenatal glucocorticoid exposure-related hypertension. In addition, the reduction of HDAC2 lowers the binding capabilities of GR and NF-kB. Furthermore, SIRT1 preserves the standard telomere length, preventing abnormal telomere lengthening in cardiovascular diseases. Another deacetylase, SIRT3, also contributes to the pathogenesis of pulmonary hypertension. Indeed, mice lacking SIRT3 develop spontaneous pulmonary arterial hypertension. In addition, the inhibitor of HDAC could help restore the damaged mitochondrial function by elevating the expression of PGC-1a and Nrf2 to resist programmed cardiovascular diseases (Higashi et al., 2016). Epidemiologic studies showed that low birth weight is associated with high cholesterol and an increased risk of cardiovascular diseases in adulthood. Prenatal nicotine exposure caused IUGR and hypercholesterolemia in male adult offspring rats. Prenatal nicotine exposure elevated serum corticosterone levels and decreased hepatic IGF1 pathway activity in male fetuses. In addition, prenatal nicotine exposure decreased the expression of SP1 and P300 enrichment and H3K27 acetylation at the promoter region of genes responsible for reverse cholesterol transport (Zhou et al., 2019).

#### Non-coding RNAs

Recently, the function of miR-101a, miR-142-3p, miR-124a, and miR-18 has been demonstrated to be involved in the regulation of GR (Anwar et al., 2016). In rat experiments, antenatal DEX exposure activated inositol 1,4,5-trisphosphate (IP3) receptor and L-type Ca^2+^ channels, especially IP3R1 and Cav1.2, which increased phenylephrine-mediated vascular contractility in offspring by epigenetic regulation of altering promoter histone modification (Xu et al., 2022). Li et al. (2022a) revealed that miRNA-mediated epigenetic regulation participated in the programming of hypercholesterolemia in female offspring after intrauterine DEX exposure. The experiment found that the application of DEX promoted glucocorticoid receptor nuclear translocation and miR-133a-3p, with the inhibition of Sirt1(101). miR-133a was critical to maintaining cardiac function and morphology and involved in cardiac hypertrophy, and Sirt1 was identified to be significant in anti-oxidative stress. Studies enhanced the evidence that maternal undernutrition increased the maternal endogenous glucocorticoids and impaired the expression of miRNAs. Glucocorticoids would induce miR-29c in a time-dependent manner to inhibit Col3A1, ELN, and MMP2, which were then direct targets of miR-29c and participated in maintaining the vascular extracellular matrix (Chuang et al., 2015). Previous research revealed that being delivered as a low birth weight (LBW) infant was associated with elevated blood pressure and adulthood cardiovascular diseases. The increased miR-449a expression suppressed *Crhr1* transcription, resulting in a higher risk of hypertension after prenatal glucocorticoid exposure. Moreover, a supplementary-enriched fat diet for LBW infants increased blood pressure by impairing the pituitary glucocorticoid negative feedback *via* miR-449a (Nemoto et al., 2020). Also, miR-122–5p was elevated after THC prenatal exposure, which inhibited the expression of IGF1R, inducing myocardial dysfunction.

Beyond miRNAs, long non-coding RNAs were also involved in the process of programmed cardiovascular diseases. Fetuses exposed to antibiotic mixtures induced postnatal downregulation of let-7a-5p (Xuan et al., 2022). Let-7a-5p had been identified as a biomarker in failing hearts that regulated the expression of insulin-like growth factor 2 mRNA-binding protein 3 (IGF2BP3), which mediated autophagy (Vilella-Figuerola et al., 2022). The inhibition of let-7a-5p increased the level of IGF2BP3 and led to more apoptosis and autophagy of cardiomyocytes in anoxia/reoxygenation (A/R) injuries (Gan et al., 2020).

## Novel therapeutic strategies in preventing programmed cardiovascular diseases

Prenatal drug exposure was associated with several fetal programmed adulthood cardiovascular diseases, including dysregulation of heart rate, hypertension, coronary artery diseases, cardiac hypertrophy, myocardial fibrosis, sensitivity to ischemic injuries, and pulmonary hypertension. As the adverse exposure could not be avoided in some circumstances, the protection or therapeutic strategies were critical to being involved in managing maternal drugs using associated programmed disorders.

Evidence revealed that maternal nutrition could program or reprogram the process of hypertension in adulthood. Previous studies indicated that a high-fat diet post drug exposure, especially glucocorticoids, would have adverse effects on the cardiovascular system, leading to more severe or earlier-onset diseases. Notably, evidence suggested that glucocorticoids, antidepressants, antiretroviral therapy, and THC-induced IUGR cause newborns to present with significantly low birth weight. High-energy feeding was considered harmful to such infants. Recently, Li et al. (2022b) demonstrated that regular nutrition feeding resulted in most offspring having a standard body weight. However, they still programmed sustainable immune activity in the heart. So, the optimal supplementary nutrition underlines its essential role in preventing programmed cardiovascular diseases.

The microbiota of offspring could be altered after prenatal antibiotic exposure. The emerging evidence demonstrated that microbiota dysregulation was involved in several diseases, especially cardiometabolic disorders. The changes in microbiota always lead to a series of substrate alterations. Previous researchers found that the reduction in SCFA was tightly associated with the onset of programmed cardiovascular diseases. Thus, the supplementary of SCFA would benefit programmed cardiovascular disease management. Acetate, the most dominant SCFA, has shown an antihypertensive effect. In addition, maternal acetate supplementation protected adult offspring against minocycline-induced hypertension by restoring the offspring’s gut microbiota and increased the expression of SCFA receptor G protein-coupled receptor 41 in the kidneys. Tain et al. (2015) revealed that melatonin prevented DEX-induced offspring hypertension and a high-fat diet by upregulating Agtr1b and Mas1 expression. In addition, the administration of melatonin could attenuate prenatal DEX-induced blood pressure increase in a rat model (Wu et al., 2014). They identified that DEX exposure decreased gene expression related to apoptosis, nephrogenesis, RAAS, and sodium transporters by increasing protein levels of HDAC1, HDAC2, and HDAC3. Melatonin therapy exerts long-term protection against neonatal DEX-induced programmed hypertension. Its beneficial effects include alterations of RAAS components and inhibition of class I HDACs.

Currently, several therapies targeting resistant oxidative stress have achieved positive results in preventing programmed cardiovascular diseases. Zhang et al. (2021) demonstrated that N-acetylcysteine blunted the programmed myocardial fibrosis and associated ventricular hypertrophy in male offspring, presenting a sex-specific manner. They identified that the administration of N-acetylcysteine would inhibit the oxidative stress, which was programmed and induced by maternal obesity. Xiao et al. (2016) performed research to identify the benefits of prenatal antioxidant therapy that protected against nicotine-induced cardiac ischemic-sensitive injuries. Nicotine was confirmed to enhance the production of ROS in the myocardium and inhibit the expression of protein kinase Cε (PKCε). The prenatal antioxidant therapy reduced ROS production and recovered the phosphorylation of glycogen synthase kinase-3β (GSK3β). Moreover, prenatal restraint stress could ameliorate the activity of AMP-activated protein kinase (AMPK) and the downstream PGC-1a/Nrf2 cascade. The impairment of AMPK/PGC-1a/Nrf2 attenuated the functional hemostasis of mitochondria, inducing cardiac hypertrophy. The restoration of PGC-1a rescued the pathological phenotype of cardiac hypertrophy. NRF2 interacted with PAK2, HO-1, or SIRT1-mediated antioxidative features in ischemic injuries or cardiotoxicity (Cao et al., 2020). Furthermore, a series of newly invented ROS scavengers, especially polyphenolic materials, show significant potential in antioxidant therapy as biomaterial candidates. The sources of natural polyphenols have been largely enriched, such as dietary fruits, vegetables, and wood (Cao et al., 2022). Epigallocatechin-3-gallate (EGCG) could induce the expression of NO production to increase endothelial cell survival (Bátori et al., 2017). Li et al. demonstrated that incorporating polydopamine nanoparticles in dextran hydrogels could efficiently eliminate the ROS at the site of infected wounds and further promote wound closure compared to the pure hydrogel group (Fu et al., 2021). In another study, Li et al. (2018) established a tannic acid (TA)-coated substrate, which could capture ROS efficiently. Thus, the TA-coated substrate promoted cellular survival under H_2_O_2_ stimulation. In this way, the natural polyphenol materials attracted significant attention.

Moreover, epigenetic regulation has been observed in the pathogenesis of programmed cardiovascular diseases after prenatal drug exposure. DNA methylation and acetylation were both involved in the process. Currently, several substrates can change the levels of methylation or acetylation, including DNMMT and HDAC inhibitors. In addition, the disorders of non-coding RNAs significantly contributed to programmed cardiovascular diseases. However, with the rapid development of viral or non-viral vectors for gene or oligonucleotide delivery, gene therapy based on non-coding RNAs presented a significant potential to restore normal cellular function. Our group used adeno-associated virus (AAV) to deliver pre-miRNA-133 in the cardiac hypertrophy mice model that could reverse the myocardial fibrosis and mitochondrial damage (Liu et al., 2022). In another research work, AAV-mediated miR-30d overexpression also attenuated cardiac hypertrophy (Li et al., 2022c).

Finally, establishing a standard for a “safety zone” on non-avoidable drug administration during pregnancy was also important. In recent research, Wada et al. (2022) evaluated the long-term effects of prenatal betamethasone administration for congenital heart block. Moreover, the results showed that the offspring were alive with normal body growth and neurodevelopment. A randomized controlled trial from McKinlay et al. (2015) revealed that exposure to repeat doses of prenatal betamethasone compared with a single course of glucocorticoids does not increase risk factors for the cardiometabolic disease at early school age. Accordingly, it would benefit the management of programmed cardiovascular diseases if more suggestions could be obtained to minimize the potential risks.

## Conclusion

The current view suggests that several cardiovascular diseases in adulthood are linked to prenatal exposure to toxins such as glucocorticoids, antibiotics, antidepressants, antiepileptics, and others. Epidemic observations and animal experiments have demonstrated that these exposures can lead to programmed cardiovascular diseases in adult offspring through various regulatory pathways. Additionally, several molecular mechanisms have been identified that contribute to the dysregulation of the cardiovascular system after prenatal exposure to drugs, including persistent oxidative stress, changes in the gut microbiota, sustained immune activity, and epigenetic malfunctions. Despite these findings, various therapeutic strategies have been proposed to prevent offspring at high risk from developing programmed cardiovascular diseases based on the molecular mechanisms uncovered.
